# Educational Strategies to Promote Adherence to Treatment in Patients with Cardiovascular Disease

**DOI:** 10.3390/ijerph19169841

**Published:** 2022-08-10

**Authors:** José Ramon Martínez-Riera, Cesar Ivan Aviles Gonzalez, Rosa Nury Zambrano Bermeo, Felice Curcio, Julián Alberto González Correa, Catalina Estrada González, Pedro Melo, Maura Galletta

**Affiliations:** 1 Department of Community Nursing, Preventive Medicine and Public Health and History of Science, University of Alicante, 03690 San Vicente del Raspeig, Spain; 2 Faculty of Health Sciences Nursing Program, Universidad Popular del Cesar, Sede Sabanas, Valledupar 200002, Colombia; 3 Department of Medical Sciences and Public Health, University of Cagliari, Cittadella Universitaria di 8 Monserrato, 09042 Cagliari, Italy; 4 School of Health, Universidad Santiago de Cali, Cali 4102, Colombia; 5 Faculty of Medicine and Surgery, University of Sassari (UNISS), Viale San Pietro 43/B, 07100 Sassari, Italy; 6 Departamento de Salud Publica, Universidad Libre de Colombia Cali, Cali 4102, Colombia; 7 Centre for Interdisciplinary Research in Health, Universidade Católica Portuguesa, 4169-005 Porto, Portugal

**Keywords:** validation studies, educational material, primary health care, health promotion, nursing

## Abstract

Introduction: Educational material is a key strategy for primary health care promotion. Purpose: To design and validate educational material adapted to the population and aimed to increase knowledge about adherence to the treatment of arterial hypertension and diabetes mellitus. Methodology: Methodological study for the design of educational material for people with diabetes mellitus and high blood pressure. For the design, content validity tests were carried out, with the participation of six experts in health education and six patients with chronic diseases. Validation was performed pursuant to the attraction, understanding, engagement, and acceptance criteria. Results: The validation confirmed that all items and criteria were above the minimal expected range. Conclusion: The design and validation of educational material provide elements that improve the education of patients about their pathologies and their adherence to treatment.

## 1. Introduction

Chronic diseases are long-lasting pathologies that evolve slowly and are frequently influenced by genetic and environmental factors, including heart diseases, infarctions, cancer, respiratory diseases, and diabetes, which are the main causes of death worldwide [1].

According to the WHO, around 41 million people die every year due to chronic diseases, which represent 71% of the worldwide mortality. Due to these diseases, 15 million people between 30 and 69 years of age die every year, and around 85% of these early deaths occur in developing countries. Among these conditions, cardiovascular diseases, such as hypertension and diabetes, are worth mentioning [2]. It is important to note that type II diabetes mellitus and hypertension are chronic diseases, unlike other cardiovascular risk factors [3,4,5].

Around 17 million people die every year due to cardiovascular diseases, which is approximately one-third of the overall population. Every year, complications of hypertension cause 9.4 million deaths; hypertension is the cause of 45% of the deaths related to cardiovascular diseases and of 51% of the deaths related to cerebrovascular accidents, and affects men and women equally. It is expected that by 2030 around 23.6 million people would die due to cardiovascular diseases [3].

Diabetes is a chronic disease whose complications include blindness, kidney failure, myocardial infarction, cerebrovascular accident, and lower-limb amputation. The prevalence of diabetes in low- and middle-income countries has increased among adults older than 18 years, evidencing a 5% increase in early mortality due to diabetes from 2000 to 2016. In 2016, this disease was the direct cause of 1.6 million deaths [6].

In Latin America and the Caribbean, 20% to 35% of the adult population suffers from hypertension. Over the last years, the number of individuals with hypertension has increased, and many of them are oblivious to their condition. In a study performed in four South American countries—Argentina, Chile, Colombia, and Brazil, only 57.1% of the adult population with high blood pressure knew that they had hypertension, which resulted in low control levels because only 18.8% of hypertensive adults controlled their blood pressure in these countries [7].

In terms of the prevalence of cardiovascular disease, Colombia is not an exception to this global situation. According to a report of the Ministerio de Salud (Ministry of Health), by 2015, hypertension had a prevalence of 7.235 in adults between 18 and 69 years who were affiliated with the Sistema de Seguridad Social en Salud (Social Security Health Care System), showing an increasing trend over the last years [8]. This could be explained by an increase in migrations from rural to urban areas [9].

Regarding type II diabetes mellitus in Colombia, the main determinants are urbanization, belonging to mestizo populations, and ageing [10]. According to the Ministerio de Salud y Protección Social (Ministry of Health and Social Protection), 3 in every 100 Colombian citizens have diabetes mellitus; however, the actual number is estimated to be higher, and 1 in every 10 people in this country are affected by this condition. By 2019, 1,294,940 people were reported to be diagnosed with diabetes, and the areas with the highest prevalence were Bogotá, Antioquia, and Valle del Cauca [11].

Considering the above, hypertension and diabetes are regarded as public health issues, which prevail worldwide and have significant effects on the patients’ life. These diseases lead to complications if their pharmacological management is inadequate or if changes in the patients’ lifestyles are insufficient [12], and they are among the main causes of care in primary health care centers [13]. Hence, patients with chronic diseases must learn to manage their diseases in order to prevent associated complications.

Primary health care centers are the first level of contact between patients, families, communities, and the health care system, enabling people to access health care near their places of residence [14]. In this context, patients with these pathologies need guidelines from healthcare professionals working at primary health care centers regarding the management of risk factors and the adoption of healthy lifestyles to improve their quality of life. Thus, they will learn self-care strategies to control their conditions and to avoid secondary and tertiary complications [15,16,17]. Moreover, community-based, behavior change interventions aimed at patients with cardiovascular diseases were found to have a positive impact on patients’ physical activity, maximum oxygen consumption, blood pressure, blood cholesterol level, and mental health [18].

For all the above, health education should consider the needs of the population, their education levels, and the individuals’ health statuses. Based on these factors, diverse educational strategies should be determined in order to raise interest in self-care and risk management. Consequently, the purpose of this study was to design and validate three educational materials (one for diabetes mellitus, two for arterial hypertension and three for adherence to pharmacological treatment) adapted to the population in order to increase knowledge about the adherence to the treatment of arterial hypertension and diabetes mellitus.

## 2. Materials and Methods

The present study corresponds to a methodological investigation where three phases were addressed: design, evaluation, and elaboration of the definitive version of the educational material, taking as a reference the guides developed by the Pan American Health Organization (PAHO) and Ziemendorff and Krause in agreement with the (PAHO/WHO) [19,20]:

(1) Design: The organization of the content, choice of messages and photographs, design of the preliminary version of the material.
(2) Evaluation: The educational material was evaluated before preparing the final version to establish its effectiveness as a didactic strategy in health education, taking into account the following criteria: attraction, understanding, involvement, and acceptance.
(3) Preparation of the definitive version of the educational material: the evaluation carried out by patients and experts was taken into account.

The sample was represented by patients who knew how to read and write (*n* = 6) and health professionals (*n* = 6). Within the inclusion criteria, it was taken into account that they were patients with chronic disease (diabetes mellitus and arterial hypertension) and health professionals who were experts in the field of health education and in the management of cardiovascular disease.

The collection of information from the group of experts and the patient was carried out through a questionnaire, for the elaboration of the questionnaire the criteria of attraction were taken into account (determines if the material draws attention), comprehension (establishes if the public understands the content and messages that are presented), involvement (assesses whether the public identifies with the messages of the educational material), and acceptance (confirms that the material does not cause discomfort in the public according to their beliefs, content, and language used), proposed by Guerra Garcia and Alva, 2003 [21]. Questions from the structured questionnaire proposed by Correa, 2014 [22], were adapted to assess the content, design, relevance, and purpose of the educational material, which allowed experts and patients to review, comment, and approve or not the content of the material; for the review, a period of 15 days was granted.

To determine content validity, the total responses per criterion were summed up, divided by the possible responses. The minimum validity index score was 0 and the maximum was 1; the cut-off point determined in the present investigation was an index of 0.7; that is, for the evaluated criteria to be valid, they had to be approved by a minimum of 70% of the evaluators. Each established criterion had two items, a total of 8 per questionnaire and the possibilities of answers were on a dichotomous scale (Yes or No).

All study phases complied with the ethical considerations for health research. The study was approved by the Ethics Committee of the School of Health and the Health Care Institution. Informed consent was obtained from all participants.

## 3. Results

Regarding the sociodemographic characteristics of the patients assessing the brochures, 33% were women and 77% were men and 33% had completed basic primary education and 77% secondary education. Regarding occupation, 50% were retired female patients, 33% were housewives, and 16% were self-employed. The age range was between 56 and 75 years, with an average age of 66 years.

Table 1 shows that the educational material assessed by patients obtained acceptable values based on the cut-off point of this test (>0.7). All items and criteria were above the expected minimum range, considering that the content of the messages sent through the material was adequate since all criteria were 100% met.

Experts assessing the brochures were health professionals, i.e., physicians and nurses with doctor’s or master’s degree or specialization in areas related to health education and management of patients with cardiovascular risk.

The findings from the expert validation suggested that the educational material obtained values that were acceptable by the technical validation performed by experts, because based on the cut-off point for this test (>0.7), all items and criteria were above the minimum expected range. The content of the messages sent through the educational material were considered appropriate since they met the attraction, understanding, engagement, and acceptance criteria with 83.3%, 75%, 91.6%, and 75% scores, respectively (Table 2).

## 4. Discussion

Regarding the results of the validation from the experts’ perspectives, all the criteria were considered valid, which is consistent with the study by Correa y Tello (2014), who suggested that all items were awarded the highest possible score by experts [23]. This shows the effectiveness of the educational material in treatment adherence for patients with chronic diseases, such as diabetes mellitus and arterial hypertension.

In this study, the criteria with the highest scores awarded by experts were engagement (91%) and attraction (80%), as opposed to the study by Herrera and Céspedes (2020), in which the attraction and engagement criteria obtained 67% and 37%, respectively [24]. This suggests that the educational material generated an educational impact on the population in terms of applicability to daily life.

Regarding the validation of the educational material by patients with diabetes mellitus and arterial hypertension, all the criteria assessed obtained the highest score, as opposed to the study by Herrera and Céspedes, in which criteria were awarded different scores [25]. This shows that the design of educational materials must consider people and their individual differences in terms of learning and plurality.

This study provides valuable elements that evidence the need for the design and validation of educational materials as a primary health care strategy, which is consistent with different research studies [25,26,27,28]. Likewise, Fernández, in his validation study of educational material for older adults with arterial hypertension, states that the material is a reliable didactic used in educational interventions to strengthen self-care and adherence to treatment [29], in this sense Ziemendorff and Krause indicate that the validation of educational material aims to understand the particularities of a specific group of people and their own characteristics, which favors the understanding and use of the educational strategy [20].

It is important to mention that there were few studies found that served as references to make comparisons with the results obtained in the present study, hence the importance of making visible the different designs and validation methods of educational material used in health education.

## 5. Conclusions

This study demonstrates the need for the design and validation of educational material intended to perform educational interventions aimed at patients with chronic disease, such as diabetes mellitus and arterial hypertension, which need to consider relevant aspects that include patients’ needs. It is possible to confirm that validation provides elements that encourage the diffusion of educational material and simplifies the use of the resources available.

## Figures and Tables

**Table 1:** Validity by patients.

Criteria		Items Evaluated	Answers per Item		Answer per Criterion		Index per Criterion	
			Yes	No	Yes	No	Yes	No
Attraction	1	Did the brochure attract your attention?	6		12		1	
	2	Do you think that colors, fonts, and images are well used in the brochure?	6					
Understanding	3	Did you understand everything you read in the brochure?	6		12		1	
	4	Were the messages clear?	6					
Engagement	5	Did you identify yourself with any of the messages in the brochure?	6		12		1	
	6	Do you think that the messages can be applied to your daily life?	6					
Acceptance	7	Do you think that the content in the brochure can be applied to your situation?	6		12		1	
	8	Do you agree with everything in the brochure?	6					

Source: Adapted from Correa, 2014.

**Table 2:** Technical validity by expert professionals.

Criteria		Items Evaluated	Answers per Item		Answer per Criterion		Index per Criterion	
			Yes	No	Yes	No	Yes	No
Attraction	1	Does the brochure attract the attention of patients with HBP and DM?	6		10	2	0.83	0.17
	2	Is the design, format, and colors appropriate for the contents?	4	2				
Understanding	3	Are the contents and messages of the brochure understandable?	5	1	9	3	0.75	0.25
	4	Do you think that the target audience will clearly understand the intention of the messages?	4	2				
Engagement	5	Do you think that the target audience will feel identified with the message?	5	1	11	1	0.91	0.09
	6	Do you think that the messages in the brochure apply to patients with HBP and DM?	6					
Acceptance	7	Do you think that the actions described in the brochure are appropriate for patients with HTA and DM?	5	1	9	3	0.75	0.25
	8	Do you think that the target audience will accept the contents of the brochure?	4	2				

Source: Adapted from Correa, 2014.

## Data Availability

Not applicable.

## References

[1] World Health Organization (2020). Chronic Diseases. https://www.who.int/topics/chronic_diseases/es/#:~:text=Las%20enfermedades%20cr%C3%B3nicas%20son%20enfermedades,del%2063%25%20de%20las%20muertes.

[2] World Health Organization (2020). Cardiovascular Diseases. https://www.who.int/cardiovascular_diseases/about_cvd/es/.

[3] Bodenheimer T., Wagner E.H., Grumbach K. (2002). Improving primary care for patients with chronic illness. J. Am. Med. Assoc..

[4] Lewanczuk R. (2008). Hypertension as a chronic disease: What can be done at a regional level?. Can. J. Cardiol..

[5] Vogeli C., Shields A.E., Lee T.A., Gibson T.B., Marder W.D., Weiss K.B., Blumenthal D. (2007). Multiple chronic conditions: Prevalence, health consequences, and implications for quality, care management and costs. J. Gen. Int. Med..

[6] World Health Organization (2020). Diabetes. https://www.who.int/topics/diabetes_mellitus/es/.

[7] PAHO Colombia (2017). World Hypertension Day 2017: Know Your Numbers. https://www3.paho.org/col/index.php?option=com_content&view=article&id=2752:dia-mundial-de-la-hipertension-2017-conoce-tus-numeros&Itemid=487.

[8] Ministerio de Salud y Protección Social de Colombia [Colombian Ministry of Health and Social Protection] (2017). Día Mundial de la Hipertensión Arterial Colombia—Mayo 17 de 2017. Ficha Técnica. [World Hypertention Day Colombia—17 May 2017. Technical Sheet]. https://www.minsalud.gov.co/sites/rid/Lists/BibliotecaDigital/RIDE/VS/PP/ENT/dia-mundial-hipertension-2017.pdf.

[9] Moya L., Morenoac J., Lombo M., Guerrero C., Aristizábal D., Vera A., Melgarejo E., Conta J., Gómez C., Valenzuela D. (2018). Expert Consensus on the Clinical Management of Arterial Hypertension in Colombia. Sociedad Colombiana de Cardiología y Cirugía [Colombian Society of Cardiology and Surgery]. https://www.sciencedirect.com/science/article/pii/S0120563318301505.

[10] Aschner P.J. (2010). Epidemiology of Diabetes in Colombia. https://www.sciencedirect.com/science/article/abs/pii/S1134323010620054.

[11] (2020). Ministerio de Salud y Protección Social de Colombia [Colombian Ministry of Health and Social Protection]. https://www.minsalud.gov.co/Paginas/Tres-de-cada-100-colombianos-tienen-diabetes.aspx.

[12] Jiménez M.L., Orkaizaguirre A., Bimbela M.T. (2015). Lifestyle and Perception of Care in Chronic Patients: Hypertension and Diabetes. http://scielo.isciii.es/scielo.php?script=sci_arttext&pid=S1132-12962015000300006.

[13] Houweling S.T., Kleefstra N., van Hateren K.J., Groenier K.H., Jong B.M., Bilo H.J. (2011). Can diabetes management be safely transferred to practice nurses in a primary care setting? A randomised controlled trial. J. Clin. Nurs..

[14] World Health Organizations Declaration of Alma-Ata International Conference on Primary Health Care, Alma-Ata, USSR, 6–12 September 1978. https://www.who.int/teams/social-determinants-of-health/declaration-of-alma-ata.

[15] Forbes A., While A. (2009). The nursing contribution to chronic disease management: A discussion paper. Int. J. Nurs. Stud..

[16] Radhakrishnan K. (2012). The efficacy of tailored interventions for self-management outcomes of type 2 diabetes, hypertension or heart disease: A systematic review. J. Adv. Nurs..

[17] Willson P.M., Brooks F., Procter S., Kendall S. (2012). The nursing contribution to chronic disease management: A case of public expectation? Qualitative findings from a multiple case study design in England and Wales. Int. J. Nurs. Stud..

[18] Lawlor E.R., Bradley D.T., Cupples M.E., Tully M.A. (2018). The effect of community-based interventions for cardiovascular disease secondary prevention on behavioural risk factors. Prev. Med..

[19] Franco-Aguilar A., Alzate-Yepes T., Granda-Restrepo D.M., Hincapié-Herrera L.M., Muñoz-Ramírez L.M. (2018). Validation of the educational material for the “Niñ@s en Movimiento” (children in motion) program to Treat child Obesity. Rev. Fac. Nac. Salud Pública.

[20] Ziemendorff S., Krause A. (2003). Guía de Validación de Materiales Educativos (con Enfoque en Materiales de Educación Sanitaria).

[21] Guerra García M., Alva M. (2003). Guía Metodológica y Video de Validación de Materiales Informativos, Educativos, Comunicacionales.

[22] Correa Tello K. (2014). Diseño y validación de material para una intervención educativa en pacientes con implante de Stent coronario. Perspect. Educ..

[23] Herrera Guerra E.P., Céspedes Cuevas V.M. (2020). Design and validation of educational material aimed at adults with Heart Failure. Rev. Cienc. Cuidad..

[24] Paradis V., Cossette S., Frasure Smith N., Heppell S., Guertin M.C. (2020). The efficacy of a motivational nurs- ing intervention based on the stages of change on self-care in heart failure patients. J. Cardiovasc. Nurs..

[25] Hernández Leiva E. (2011). Epidemiología del síndrome coronario agudo y la insuficiencia cardiaca en Latinoamérica. Rev. Esp. Cardiol..

[26] Otsu H., Moriyama M. (2011). Effectiveness of an educational self-management program for outpatients with chronic heart failure. Jpn. J. Nurs. Sci..

[27] Bartolomé A., Cabero J.E. (1999). El Diseño y Producción de Medios Para la Enseñanza.

[28] Liévano Fiesco M., García Londoño G., Leclercq Barriga M., Liévano De Lombo G., Solano Salazar K. (2009). Validación del material lúdico de la estrategia educativa basada en juegos para la promoción de estilos de vida saludable en niños de cuatro a cinco años de edad. Universitas Scientiarum.

[29] Fernández A., Manrique F.G. (2010). Efecto de la intervención educativa de enfermería en la agencia de autocuidado del adulto mayor hipertenso de Boyacá, Colombia, Suramérica. Cienc. Enfermería.

